# A Lightweight Key Agreement Protocol for V2X Communications Based on Kyber and Saber

**DOI:** 10.3390/s25226938

**Published:** 2025-11-13

**Authors:** Yinfei Dai, Qi Wang, Xiao Song, Shaoqiang Wang

**Affiliations:** 1 College of Computer Science and Technology, Changchun University, Changchun 130022, China; 2 College of Computer Science and Technology, Jilin University, Changchun 130012, China

**Keywords:** post-quantum cryptography, vehicular networks, key agreement, Kyber, Saber

## Abstract

**Highlights:**

**What are the main findings?**

- A hybrid post-quantum key agreement protocol is proposed for V2X environments, combining Kyber and Saber to enhance both security and computational efficiency.
- The protocol achieves authenticated key exchange with low handshake latency, verified through Veins + SUMO simulations, and integrates algorithm-level optimizations for embedded platforms to enhance efficiency and adaptability.

**What are the implications of the main findings?**

- The protocol shows high adaptability to dynamic and low-power vehicular environments, supporting secure and efficient key exchange under quantum threats.
- It provides a practical and forward-compatible cryptographic framework for intelligent transportation systems in the post-quantum era.

**Abstract:**

This paper proposes a post-quantum secure key agreement protocol tailored for vehicular networks (V2X), addressing the dual challenges of quantum resistance and lightweight deployment. The hybrid scheme integrates two lattice-based Key Encapsulation Mechanisms (KEMs)—Kyber and Saber—to construct a dual-path handshake framework that enhances cryptographic redundancy and ensures robustness against quantum attacks. The protocol achieves secure and authenticated key exchange through RSU public-key broadcasting, OBU dual-path encapsulation, and session-key derivation using HMAC and timestamps. To support efficient execution in embedded vehicular environments, several algorithm-level optimizations are incorporated, including Number Theoretic Transform (NTT) acceleration for Kyber, AVX2-based parallelism for Saber, and integer inner-product techniques to minimize computational overhead. Experimental validation on a Veins + SUMO vehicular simulation platform demonstrates that the proposed protocol reduces handshake latency by nearly 60% compared with RSA, achieves delay performance comparable to ECDH, and lowers total resource consumption by around 40%. These results confirm that the Kyber + Saber hybrid protocol provides a practical, scalable, and quantum-resistant solution for secure V2X communication in dynamic, resource-constrained, and latency-sensitive environments.

## 1. Introduction

### 1.1. Background and Research Motivation

With the rapid development of Intelligent Transportation Systems (ITS), Vehicle-to-Everything (V2X) communication has become a foundation for autonomous driving, traffic efficiency, and smart cities. V2X enables interactions among vehicles (V2V), roadside units (V2I), pedestrians (V2P), and cloud platforms (V2C), supporting cooperative awareness, collision avoidance, and dynamic routing.

The key entities are On-Board Units (OBUs) and Roadside Units (RSUs). OBUs handle authentication and secure message exchange on resource-constrained embedded hardware, while RSUs possess higher computational and broadcasting capability to connect multiple OBUs. These OBU–RSU interactions feature high mobility and strict latency limits, imposing strong requirements on lightweight and efficient security protocols.

**Figure 1:**
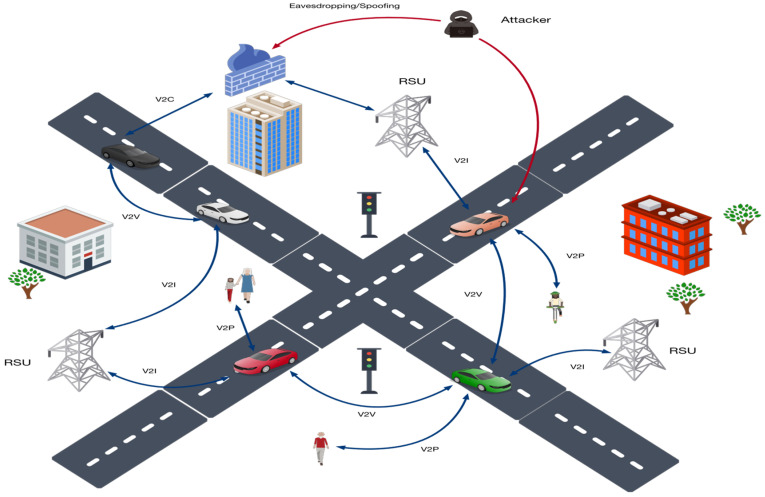
Typical V2X communication architecture and attack surfaces.

Figure 1 illustrates a typical V2X architecture involving OBUs, RSUs, cloud servers, and pedestrian devices. It also highlights potential attack points—eavesdropping, spoofing, and man-in-the-middle—that threaten confidentiality, integrity, and authenticity in vehicular communications.

As illustrated in Figure 1, the V2X network is inherently open and dynamic, enabling adversaries to exploit multiple communication links. Typical threats include identity forgery and false data injection in V2V communication, eavesdropping and protocol downgrade attacks in V2I interactions, spoofing of pedestrian devices in V2P scenarios, and data theft or tampering in V2C uplinks. Such attacks compromise the system’s trust foundation and pose severe risks to traffic safety [1]. Therefore, ensuring the confidentiality, integrity, and authenticity of all V2X communication links is a fundamental goal in protocol design.

At present, most secure communication solutions for V2X rely on Public Key Infrastructure (PKI) systems built upon RSA or ECC [2,3,4,5]. However, the advent of quantum computing—particularly Shor’s algorithm, which can efficiently break RSA and ECC [6]—makes these classical schemes insecure in the medium to long term. Consequently, Post-Quantum Cryptography (PQC) has become a promising foundation for developing the next generation of secure and quantum-resistant V2X communication protocols [7].

In recent years, the research community has increasingly explored the integration of lattice-based cryptography into V2X protocol design, achieving notable progress particularly in authentication and privacy preservation. Shahidinejad et al. proposed an anonymous lattice-based authentication protocol for vehicular communications [8], while Nath et al. developed the LbPV scheme to realize privacy-preserving mutual authentication based on lattice cryptography [9]. These studies have laid a solid theoretical foundation for post-quantum secure communications; however, most existing approaches remain focused on identity authentication and have not yet achieved low-latency performance or hybrid KEM optimization in the key agreement phase.

Among the PQC candidates, Kyber (based on the Learning With Errors, LWE, problem) and Saber (based on the Learning With Rounding, LWR, problem) are particularly suitable for embedded devices in V2X environments, as they combine strong security guarantees with relatively low computational overhead [10]. Both algorithms have been included in the NIST PQC standardization process, providing a solid foundation for constructing quantum-resistant, lightweight, and efficient key agreement protocols [11,12].

### 1.2. Encryption Requirements and Protocol Challenges in V2X

In vehicular networks (V2X), security and real-time performance must be jointly maintained. High-speed vehicles exchange short-lived messages with surrounding nodes under strict latency and power constraints, making lightweight cryptographic operations essential. Traditional public-key schemes such as RSA and ECDH impose heavy computation and long handshake delays on ARM-based embedded devices that lack hardware accelerators, and they offer limited support for fast session recovery in dynamic network topologies. Moreover, their mathematical foundations can be broken by quantum algorithms such as Shor’s, undermining long-term security. Future V2X key agreement protocols must therefore achieve efficient computation on constrained hardware, minimize message size and handshake rounds for rapid reconnection, and ensure post-quantum resistance based on LWE and LWR problems. To meet these goals, this paper proposes a hybrid key-encapsulation mechanism combining Kyber and Saber, balancing quantum security, computational efficiency, and lightweight deployment to provide a practical and forward-looking solution for secure V2X communications.

### 1.3. Contributions of This Work

To address the growing quantum security challenges in vehicular networks (V2X), this paper proposes a lightweight hybrid key agreement protocol integrating lattice-based post-quantum cryptography. By combining Kyber and Saber, the protocol achieves a balanced trade-off among security, latency, and resource efficiency, explicitly tailored to the high-mobility and low-latency requirements of V2X systems.

The proposed design employs a dual-path Hybrid Key Encapsulation Mechanism (Hybrid-KEM) architecture, in which Kyber (LWE-based) ensures strong cryptographic robustness, while Saber (LWR-based) serves as a lightweight auxiliary module to accelerate session initialization and reduce computational load. A coordinated pre-authentication and fast-handshake mechanism further minimizes communication and computation overhead while maintaining robustness, effectively overcoming the performance limitations of single-algorithm schemes. To accommodate frequent, short-lived vehicular connections, the protocol optimizes handshake rounds, message formats, and parameter encoding, thereby reducing on-board unit (OBU) processing costs and improving overall responsiveness. Additionally, a lightweight session resumption strategy is introduced to enhance reliability under dynamic link conditions, maintaining millisecond-level handshake latency that complies with the typical 10 ms V2X timing constraint.

The protocol is implemented and evaluated on the Veins + SUMO vehicular network simulation platform. Experimental results demonstrate that, compared with classical RSA and ECDH protocols, the proposed Hybrid-KEM scheme reduces handshake latency by nearly 60% and overall resource consumption by approximately 40%, confirming its suitability for resource-constrained vehicular environments. Built upon Kyber and Saber—both NIST PQC finalists with proven IND-CCA2 security—the proposed protocol further integrates timestamp- and nonce-based HMAC authentication [13] to ensure message integrity, forward secrecy, and resistance against replay, man-in-the-middle, and quantum attacks, all while maintaining high communication efficiency.

Overall, this work establishes a comprehensive and practical framework encompassing protocol architecture design, lightweight optimization, simulation-based performance evaluation, and formal security analysis. The proposed Kyber + Saber hybrid scheme provides a scalable, deployable, and quantum-resistant key agreement solution for next-generation 5G/6G-enabled vehicular networks, contributing a valuable foundation for future secure vehicular communication systems.

## 2. Related Work and Background

### 2.1. V2X Communication and Security Requirements

Vehicle-to-Everything (V2X) communication, including Vehicle-to-Vehicle (V2V) and Vehicle-to-Infrastructure (V2I) interactions, serves as the cornerstone of Intelligent Transportation Systems (ITS). It enables critical applications such as cooperative collision avoidance and real-time traffic updates. These applications impose stringent requirements on security protocols, which must ensure low latency, high reliability, and strong identity authentication to resist attacks such as spoofing, replay, and man-in-the-middle.

Traditional V2X key agreement protocols typically rely on Public Key Infrastructure (PKI) and algorithms such as RSA and ECDSA. However, the emergence of quantum computing poses an existential threat to these systems. Shor’s algorithm can efficiently solve the underlying hard problems (integer factorization and discrete logarithms), rendering them vulnerable in the quantum era.

### 2.2. Post-Quantum Cryptography and the Foundations of KEM

Post-Quantum Cryptography (PQC) aims to develop cryptographic systems resistant to both classical and quantum adversaries. From a mathematical perspective, PQC has been systematically studied [14], with lattice-based cryptography rapidly emerging as the mainstream approach over the past decade [15].

The Key Encapsulation Mechanism (KEM) has become a fundamental primitive for PQC-based key exchange. A typical KEM consists of three algorithms:

Key Generation: (pk,sk)←KeyGen(), which outputs a public key pk and a secret key sk.

Encapsulation: (ct,ss)←Encaps(pk), which, given a public key pk, produces a ciphertext ct and a shared secret ss.

Decapsulation: ${ss}^{\prime }\leftarrow Decaps(sk,ct)$, which, given a secret key sk and ciphertext ct, recovers the shared secret ${ss}^{\prime }$.

Correctness requires $ss={ss}^{\prime }$. As the core building block of many NIST PQC finalists, KEM provides a robust framework for constructing quantum-resistant key agreement schemes [16].

### 2.3. Lattice-Based KEMs: Kyber and Saber

Lattice-based cryptography has gained prominence due to its strong security foundations and practical efficiency, making it a leading candidate in the NIST PQC competition.

Kyber: Based on the hardness of the Module-LWE (MLWE) problem, Kyber achieves strong IND-CCA2 security. It leverages the Number Theoretic Transform (NTT) to accelerate polynomial multiplications, enabling efficient performance in mainstream applications [17]. Although some studies have proposed refined lattice attacks [18], the LWE problem is still widely considered intractable for quantum adversaries. Variants such as Ring-LWE-based KEMs have also been introduced to further reduce ciphertext size [19].

Saber: Based on the Module-LWR (MLWR) problem, Saber replaces explicit noise sampling with deterministic rounding operations. This design typically yields higher efficiency in hardware and embedded implementations, making Saber particularly suitable for resource-constrained devices such as OBUs [20].

Both Kyber and Saber have been extensively optimized—for instance, using AVX2 vectorization and compact sampling techniques such as CDT [21,22]—to achieve high performance on embedded platforms. These optimizations demonstrate their feasibility for deployment in V2X environments [23,24].

Both Kyber and Saber are lattice-based post-quantum key encapsulation mechanisms (KEMs) selected in the NIST PQC standardization process, exhibiting complementary strengths in security and computational efficiency.

Kyber, based on the Module Learning With Errors (Module-LWE) problem, provides strong theoretical guarantees and robust mathematical foundations, making it ideal as the main security primitive. It offers high quantum resistance and moderate key sizes but involves higher computational costs due to polynomial operations.

Saber, derived from the Module Learning With Rounding (Module-LWR) problem, replaces explicit noise sampling with deterministic rounding, significantly reducing the cost of random number generation and polynomial multiplication. This design enables superior efficiency in embedded and energy-constrained environments.

Considering the heterogeneity of vehicular devices such as OBUs and RSUs—with notable differences in computing power and energy budgets—a single algorithm cannot simultaneously satisfy both real-time and security requirements. To address this, the proposed Hybrid-KEM combines Kyber for long-term robustness and Saber for fast response and low latency, ensuring post-quantum resistance while maintaining deployability on constrained vehicular hardware.

In this collaborative design, Kyber secures the session key against quantum attacks, while Saber enhances efficiency under dynamic link conditions, achieving high security, low latency, and embedded adaptability. Prior benchmarking by Fitzgibbon et al. validated the feasibility of Kyber and Saber on ARM-based platforms [25], and Wang et al. demonstrated improved robustness of post-quantum KEMs under lossy and interfered network environments [26]. These studies provide the experimental basis for the hybrid mechanism adopted in this work.

### 2.4. PQC in V2X: Current Status and Research Gaps

Research on integrating post-quantum cryptography (PQC) into vehicular networks (V2X) has progressed mainly along two directions: algorithm optimization and protocol integration. In terms of algorithm optimization, Kyber and Saber have been efficiently ported to embedded platforms such as ARM Cortex-M, RISC-V, and ESP32, achieving latency and power consumption suitable for vehicular applications. On the protocol side, several studies have integrated PQC-based KEMs into frameworks like TLS 1.3, achieving handshake delays comparable to or even lower than classical ECDHE [27]. Early research focused on single KEM–based authenticated key exchange schemes [28,29], while later work proposed PQC-enabled group key agreement and secure channel establishment tailored for V2X systems [30].

Despite these advances, notable gaps remain. Most existing studies rely on a single algorithm or conduct isolated performance benchmarks, lacking a holistic, protocol-level architecture that leverages the complementary advantages of multiple PQC schemes to cope with the dynamic, heterogeneous nature of V2X environments. Key challenges include the absence of hybrid designs that combine Kyber’s strong security with Saber’s efficiency [31], insufficient adaptability to V2X-specific conditions such as frequent link disruptions and high concurrency [32], and a lack of comprehensive comparative evaluations between PQC-based and classical RSA/ECDH protocols under realistic vehicular scenarios [33].

At present, most vehicular network security communication protocols are still built upon traditional public key infrastructures (PKIs). For instance, Dedicated Short Range Communications (DSRC) adopts an ECC-based certificate authentication mechanism, while LTE-V2X protocols continue to rely on RSA/ECC-based digital signature frameworks. Although such systems can provide reliable authentication and data encryption under classical computational assumptions, their security faces fundamental threats in the quantum computing era. Quantum algorithms, such as Shor’s algorithm, are capable of solving integer factorization and elliptic curve discrete logarithm problems in polynomial time, which would lead to rapid key disclosure and signature forgery, thereby rendering traditional PKI schemes completely insecure.

In addition, conventional PKI models exhibit significant performance bottlenecks in highly dynamic and high-frequency vehicular environments. Certificate verification and revocation list synchronization require frequent communication and computational overhead, making it difficult to meet the millisecond-level handshake latency requirements of V2X systems. Particularly in dense traffic scenarios with multiple simultaneous vehicle connections or RSU clusters, the centralized authentication structure can easily become a system bottleneck, resulting in congestion and cumulative authentication delays.

Therefore, with the rapid advancement of quantum computing and the continuous expansion of V2X network scale, traditional RSA/ECC-based security architectures can no longer simultaneously satisfy both security and real-time performance requirements. It has become imperative to introduce lattice-based post-quantum cryptographic algorithms to provide long-term, sustainable security protection for future vehicular networks.

### 2.5. Summary of Security Requirements for V2X

This chapter reviewed the security requirements of V2X communications and highlighted the necessity of adopting PQC. It introduced promising lattice-based KEM algorithms—Kyber and Saber—and discussed their optimization for embedded systems. Despite significant progress, there remains a clear gap in designing flexible, hybrid protocol architectures tailored to the demanding conditions of V2X. This gap motivates our proposal of a novel hybrid KEM protocol in the next chapter.

## 3. Protocol Design and Construction

To address the requirements of low latency, high security, and post-quantum resistance in vehicular communications, this study proposes a lightweight key agreement protocol based on a hybrid key encapsulation mechanism (Hybrid-KEM) that integrates Kyber and Saber. The protocol adopts a dual-KEM parallel negotiation design, combining the quantum-resistant security of Kyber with the lightweight efficiency of Saber, and is tailored for secure communication between On-Board Units (OBUs) and Road-Side Units (RSUs) in V2X scenarios.

### 3.1. Design Objectives

To cope with the security challenges faced by V2X in the era of quantum computing, while ensuring real-time performance and resource efficiency, the proposed key agreement protocol adheres to the following five design objectives.

Quantum Resistance: The protocol leverages post-quantum secure Kyber (based on LWE) and Saber (based on LWR). By deploying both algorithms in parallel, it achieves quantum resistance with added redundancy.

Low-Latency Communication: A three-step handshake process is adopted—pre-broadcasting of public keys, dual ciphertext submission, and single-response authentication. This, combined with parallel processing, reduces overall handshake delay.

Resource-Constrained Adaptability: The protocol features computational minimalism, relying solely on polynomial multiplication and modular arithmetic. It is designed for efficient execution on common embedded platforms (such as Raspberry Pi, ESP32, and ARM Cortex-M series microcontrollers), ensuring seamless integration and compatibility with typical hardware architectures in V2X systems.

Robustness via Hybrid-KEM: Kyber and Saber operate in parallel, with their outputs combined through a key derivation function (KDF). Even if one algorithm fails, the overall protocol security remains intact.

Authentication and Integrity: During key agreement, the protocol integrates HMAC, identity identifiers, and timestamps to ensure trustworthy authentication and prevent replay and man-in-the-middle attacks.

#### 3.1.1. V2X Security Scenario and Trust Model

To describe the proposed protocol clearly, this study defines the trust and security model adopted for vehicular network (V2X) communications. A centralized trusted root entity, the Certification Authority (CA), manages long-term identity credentials and issues digital certificates to all participants. Operating within the core network of transportation authorities or service providers, the CA is only involved during registration and certificate renewal, not in each key exchange process.

To reduce the computational and communication load on the CA, regional key and certificate distribution nodes are introduced. These nodes periodically synchronize the latest certificate revocation lists (CRL), identity information, and post-quantum public-key parameters from the CA, distributing them to all RSUs within their region. This structure maintains a unified root of trust while enabling RSUs to perform fast local authentication without constant communication with the central CA.

Each Road-Side Unit (RSU) acts as both a communication relay and a local authentication proxy. During initialization, the RSU receives post-quantum key pairs (Kyber and Saber) and identity credentials from the CA or a regional center, broadcasting its signed identity and public keys to nearby vehicles. Vehicles equipped with the CA’s root key can verify the RSU’s authenticity locally. On the vehicle side, each On-Board Unit (OBU) completes registration during manufacturing or its first connection, obtaining a certificate and cryptographic parameters from the CA. Because OBUs often operate offline, they rely on cached root keys and RSU broadcasts for local verification.

Based on this trust model, the proposed Kyber + Saber lightweight key agreement protocol focuses on establishing a quantum-secure session key between RSU and OBU within milliseconds, assuming mutual authentication has already been verified. The CA ensures identity validity, while the proposed protocol guarantees communication efficiency and post-quantum security. This layered design achieves a balance between engineering deployability and cryptographic strength, making the scheme easily integrable into existing vehicular PKI frameworks.

#### 3.1.2. Hybrid Post-Quantum PKI Deployment Scheme

With the rapid advancement of quantum computing, traditional RSA- and ECC-based Public Key Infrastructure (PKI) systems are gradually losing their security foundations. Since vehicular communication frameworks such as IEEE 1609.2 [34] and ETSI ITS-SC [35] are already widely deployed, a complete replacement of the existing PKI is impractical and would cause major compatibility issues. Instead of redesigning the infrastructure, this study introduces a post-quantum key exchange mechanism within the existing vehicular PKI, enabling a smooth transition from classical to quantum-resilient security.

The proposed architecture retains the CA as the root of trust, preserving existing certificate issuance, identity registration, and revocation procedures. During communication, OBUs and RSUs establish quantum-secure session keys using the Kyber + Saber hybrid key encapsulation mechanism (Hybrid-KEM), while identity verification during registration can still rely on classical signatures such as ECDSA or coexist with post-quantum signature schemes for backward compatibility. In this hybrid PKI framework, the traditional trust management and certificate validation processes remain unchanged, while only the key-exchange module is upgraded, significantly enhancing quantum resistance without disrupting current operations.

This hybrid design ensures compatibility, as post-quantum upgrading can be achieved simply by updating algorithm parameters without altering CA or certificate structures. It also supports gradual deployment, allowing classical and post-quantum devices to coexist during transition, and maintains scalability for future integration of other NIST-standardized PQC algorithms. Overall, the Kyber + Saber protocol functions as a lightweight post-quantum extension module that fits seamlessly into existing vehicular PKI frameworks, providing long-term quantum-resilient security and a practical evolution path for large-scale deployment.

### 3.2. Protocol Architecture Overview

**Figure 2:**
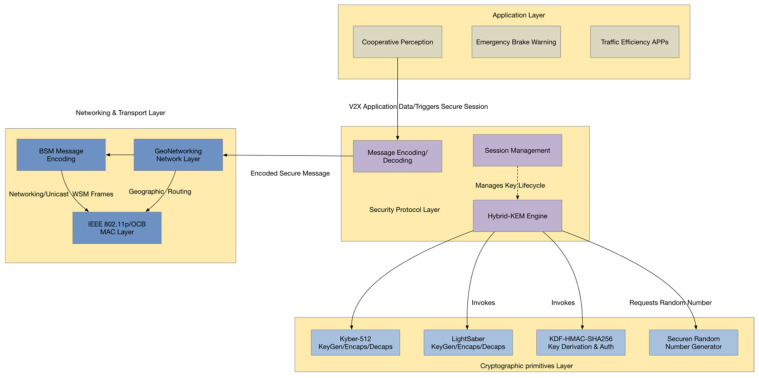
System Layered Architecture and Data Flow Diagram.

To meet the combined requirements of quantum-resistant security, computational efficiency, and low-latency adaptability in vehicular network (V2X) communications, this paper proposes a three-phase key agreement protocol architecture. As shown in Figure 2, the system enables lightweight key synchronization between the RSU and OBU, integrating Kyber and Saber—two post-quantum key encapsulation mechanisms (KEMs)—with an HMAC-based authentication scheme to construct a secure, efficient, and easily deployable hybrid post-quantum key agreement framework.

Previous studies have explored Kyber-based and HMAC-assisted quantum-resistant authentication mechanisms in mobile environments such as unmanned aerial vehicle (UAV) communications, demonstrating their feasibility and security in low-latency wireless scenarios [36]. Building upon these foundations, the proposed Kyber + Saber hybrid design extends the concept to V2X environments, achieving parallel encapsulation and dual-algorithm cooperation to handle higher mobility and more complex network dynamics.

Before protocol execution, a registration phase managed by the Certification Authority (CA) establishes system trust. The CA assigns long-term identity key pairs and digital certificates containing device identifiers, algorithm parameters, and validity periods for each entity. Both OBUs and RSUs register their identities and cryptographic materials with the CA or regional center, after which the CA’s root public key is preloaded on all devices for offline verification of RSU broadcasts. This registration occurs only once—during initialization or first onboarding—allowing subsequent communications to operate without direct CA involvement.

During operation, authenticated OBUs and RSUs perform the proposed three-phase key agreement procedure: initialization and broadcasting, parallel key encapsulation and exchange, and session key derivation and authentication. This design minimizes communication overhead while maintaining strong security and scalability for dynamic vehicular environments.

**Figure 3:**
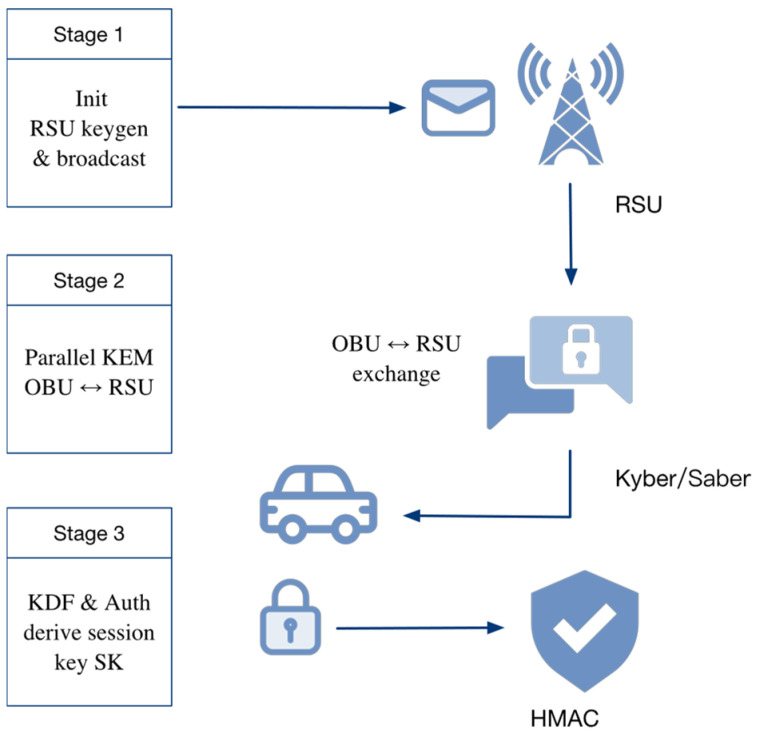
Three-Phase V2X Key Agreement Process Based on Kyber and Saber.

As shown in Figure 2, the proposed protocol adopts a four-layer architecture consisting of the application, security, network, and cryptographic layers. V2X applications such as cooperative perception and emergency braking initiate communication requests that trigger the secure session setup. The security protocol layer forms the system core, integrating message encoding, a hybrid-KEM engine, and session management to perform key agreement and maintain a secure context. Processed messages are encapsulated at the network and transport layer into IEEE 802.11p [37]/OCB-compliant frames for transmission and routing. The cryptographic primitives layer provides essential support for upper layers, including Kyber, Saber, KDF-HMAC, and secure random number generation. Well-defined interfaces ensure seamless data exchange and modular implementation across layers, forming a complete protocol stack. The overall interaction process consists of three phases, as illustrated in Figure 3.

Stage 1: Initialization and Broadcast

The Road-Side Unit (RSU) generates its own key pairs (Kyber and Saber) and periodically broadcasts the public keys together with identity information within its coverage area. These broadcasts allow On-Board Units (OBUs) to retrieve and locally cache the information. In Figure 3, the upper-left “Stage 1” block denotes the key initialization and broadcast process, while the antenna icon represents the RSU’s broadcast operation.

Stage 2: Parallel Key Encapsulation and Exchange

When an OBU enters the communication range of an RSU, it reads the broadcast public keys and initiates parallel key encapsulation based on both Kyber and Saber. The OBU then generates the corresponding ciphertexts and sends them to the RSU, forming the first handshake message. In Figure 3, the central “OBU ↔ RSU exchange” arrow illustrates this data transfer, with the Kyber/Saber icons denoting the cryptographic primitives employed.

Stage 3: Session Key Derivation and Authentication

Upon receiving the ciphertexts, the RSU decapsulates them using its private keys to recover the shared secret material. It then invokes a Key Derivation Function (KDF) to derive the final symmetric session key, denoted as $K_{final}$. Subsequently, the RSU generates an authentication response using HMAC and sends it back to the OBU, thereby completing identity verification and key synchronization. In Figure 3, the lock and shield icons represent session key generation and HMAC-based authentication, respectively.

Unlike conventional bidirectional handshake protocols, the proposed design adopts a unidirectional authentication response mechanism, which completes key agreement in only two message exchanges. This significantly reduces communication latency and is particularly suitable for V2X scenarios characterized by frequent link disruptions and short-lived connection windows.

### 3.3. Protocol Procedure and Cryptographic Operations

To meet the combined requirements of low latency, strong security, and lightweight computation in V2X, the proposed protocol realizes a hybrid key agreement through a carefully designed three-stage mechanism. The following sections provide a detailed description and formalization of the operations performed in each stage, including cryptographic operations, message formats, and verification procedures.

#### 3.3.1. Algorithm Setup and Assumptions

To build a lightweight yet post-quantum secure key-agreement protocol for V2X, we unify the notation, cryptographic building blocks, and interface conventions used throughout the protocol. These definitions ensure clarity and implementability of the protocol logic.

1. Base algorithms and notation

The cryptographic primitives employed in this work are configured as follows. Kyber512 is adopted as the parameter set for the Kyber scheme, while LightSaber, the lightweight variant of Saber, is selected to improve suitability for resource-constrained embedded platforms. The hash function H() is instantiated with SHA-256, which also underpins the Key Derivation Function (KDF) (e.g., HKDF-SHA256) for deriving the final symmetric session keys. For message authentication, the protocol employs HMAC-SHA256 to ensure data integrity and authenticity. System entities are represented by unique identifiers, namely ${ID}_{OSU}$ for the On-Board Unit (OBU) and ${ID}_{RSU}$ for the Road-Side Unit (RSU). Furthermore, timestamps are incorporated to guarantee freshness and provide replay resistance, where $\;T_{1}$ denotes the handshake initiation time and $T_{2}$ represents the handshake response time.

2. KEM interface definitions

We model each Key Encapsulation Mechanism (KEM) with the following standard interface that applies to both Kyber and Saber:

Key generation: (pk,sk)←KeyGen()—outputs a public key pk and a secret key  sk.

Encapsulation: (ct,ss)⟵Encaps(pk)—given a recipient public key pk, outputs a ciphertext ct and a shared secret ss.

Decapsulation: ${ss}^{\prime }\leftarrow Decaps(sk,ct)$—given a secret key sk  and a ciphertext ct, recovers the shared secret ${ss}^{\prime }$.

Correctness requires ss=ss′ for matching encapsulation/decapsulation pairs.

In the proposed protocol, both Kyber and Saber implement the above interface and are run in parallel during the encapsulation phase; their recovered shared materials are later combined (via the KDF) to form the final symmetric session key.

Security and implementation assumptions. We assume that: (i) private keys sk are securely stored and never exposed; (ii) a cryptographically secure random number generator is available for key generation and any nonce/entropy needs; and (iii) the underlying implementations of Kyber512 and LightSaber follow their respective specification best practices (padding, parameter choices, constant-time operations where required).

3. Kyber Algorithm Workflow

Kyber is a Key Encapsulation Mechanism (KEM) based on the Module-Learning With Errors (MLWE) problem. Its core idea is that the value

t=A · s+e  mod q

computed from a public matrix A and a secret vector s, appears computationally indistinguishable from random to an adversary who does not know s. This property provides the foundation for public-key cryptographic security.

In this work, the Kyber512 parameter set is adopted as a reference, where

q=3329  (modulus)

n=256  (polynomial dimension)

k=2 (module dimension)

$\eta_{1}=3\;$(parameter for the centered binomial distribution used to sample secret and error vectors)

A random seed is used to deterministically generate the public matrix A.

The Kyber scheme consists of Key Generation (KeyGen), Encapsulation (Encaps), and Decapsulation (Decaps), as illustrated in Algorithm 1.
**Algorithm 1.** Kyber Algorithms(a) Key Generation1.$seed\leftarrow U({\left\{0,1\right\}}^{\lambda })$2.$A\leftarrow Gen(seed)\in R_{q}^{k\times k}$3.$s_{i}\leftarrow {CBD}_{\eta_{1}},e_{i}\leftarrow {CBD}_{\eta_{1}}$4.$s=\left(s_{1},s_{2},\ldots ,s_{k}\right)\in R_{q}^{k}$5.$e=(e_{1},e_{2},\ldots ,e_{k})\in R_{q}^{k}$6.Check(e): $if\;\;\;TotalError=\sum_{i=1}^{k}\sum_{j=0}^{n-1}\left|e_{i}\left[j\right]\right|>L$ Return false Else Return ture7.t=A · s+e  mod q8.$return\;\left({pk}_{k}=\left(seed,t\right),{sk}_{k}=s\right)$(b) Encapsulation1.Input:pk=(A,t)2.$m\leftarrow U({\left\{0,1\right\}}^{\lambda }$3.$r\leftarrow CBD{\left(\eta_{1}\right)}^{k}$4.u=A⋅r mod q5.$v=t\cdot r+Encode\left(m\right)+e^{\prime }\;mod\;q$6.${ct}_{k}=(u,v)$7.${ss}_{k}=KDF(u||v||m)$8.$return\left({ct}_{k},{ss}_{k}\right)$(c) Decapsulation1.$Input:{sk}_{k}=s,{ct}_{k}=(u,v)$2.$m^{\prime }=v-s\cdot u\;mod\;q$3.$CheckError(m^{\prime })\to True/False$4.${ss}_{k}=KDF(u||v||m^{\prime })$5.$return{\;ss}_{k}$

To ensure stability in the decapsulation phase, Kyber introduces an Error Checking Function to validate whether the error vector e remains within the acceptable range. Specifically, the function computes:

$$TotalError=\sum_{i=1}^{k}\sum_{j=0}^{n-1}\left|e_{i}[j]\right|$$

If the result exceeds the predefined threshold L, the sample is considered invalid and the process is restarted; otherwise, the computation continues.

Moreover, Kyber’s randomness relies on the Centered Binomial Distribution (CBD). Both secret keys and error vectors are sampled using parameter $\eta_{1}$. The final shared secret is derived using a Key Derivation Function (KDF), ensuring uniqueness and forward secrecy of session keys across multiple rounds of communication.

4. Saber Algorithm Flow

Saber is a lightweight KEM based on the Module-Learning With Rounding (MLWR) problem. Unlike Kyber, Saber employs deterministic rounding operations instead of noise sampling in certain steps, thereby reducing computational overhead and power consumption. This makes Saber particularly suitable for deployment in resource-constrained vehicular networks (V2X terminals).

In this work, the LightSaber parameter set is adopted as a reference, with the following parameters:

q=3329  (modulus)

h=2 (number of public key matrix columns)

n=256 (polynomial dimension)

A random seed is used to initialize the pseudorandom generator for constructing the public matrix A.

The Saber scheme includes Key Generation (KeyGen), Encapsulation (Encaps), and Decapsulation (Decaps), as shown in Algorithm 2.
**Algorithm 2.** Saber Algorithms(a) Key Generation1.$seed\leftarrow U({\left\{0,1\right\}}^{\lambda })$2.$A\leftarrow Gen(seed)\in R_{q}^{h\times n}$3.$s_{i}\leftarrow {CBD}_{\eta_{1}},e_{i}\leftarrow {CBD}_{\eta_{1}}$4.$s=(s_{1},s_{2},\ldots ,s_{k})\in R_{q}^{k}$5.$e=(e_{1},e_{2},\ldots ,e_{k})\in R_{q}^{k}$6.b=A · s+e  mod q7.$return\left({pk}_{s}=\left(A,b\right),{sk}_{s}=s\right)$(b) Encapsulation1.$Input:{pk}_{s}=(A,b)$2.$m\leftarrow U({\left\{0,1\right\}}^{\lambda })$3.$r_{s}\leftarrow {CBD}_{\eta_{2}}$4.$u_{s}=A\cdot r_{s}\;mod\;q$5.$v_{s}=t_{s}\cdot r_{s}+Encode\left(m_{s}\right)+e_{s}^{\prime }\;mod\;q$6.${ct}_{k}=(u,v)$7.${ss}_{s}=KDF(u_{s}|\left|v_{s}\right||m_{s})$8.$return\;({ct}_{s},{ss}_{s})$(c) Decapsulation1.$Input:{sk}_{s}=s,{ct}_{s}=\left(u_{s},v_{s}\right)$2.$m_{s}^{\prime }=v_{s}-s\cdot u_{s}\;mod\;q$3.${ss}_{s}=KDF(u_{s}|\left|v_{s}\right||m_{s})$4.$Return\;{ss}_{s}$

During KeyGen, the public key matrix A is generated from the seed, and secret and error vectors are sampled from the centered binomial distribution. The public key is then computed as:

b = A · s + e  mod q

Compared with Kyber, Saber incorporates an approximate integer inner product optimization, replacing traditional floating-point multiplications. This approach enhances compatibility with embedded platforms while reducing power consumption.

During Encapsulation, given the public key ${pk}_{s}=(A,b)$ and a random message $m_{s}$, the ciphertext $\left(u_{s},v_{s}\right)$ is generated, and the shared secret ${ss}_{s}$ is derived via the KDF. During Decapsulation, the receiver uses the private key s  to recover an estimated message $m_{s}^{\prime }$ and recompute the shared secret ${ss}_{s}$.

5. Error Check Function checkE(e)

To further guarantee correctness during the Decapsulation phase, both Kyber and Saber introduce an Error Checking Mechanism. The function CheckE(e) ensures that the sampled error vector does not exceed the predefined tolerance, thereby preventing decryption failures or incorrect key recovery.

In this work, the tolerance threshold is set to L. The function sums the absolute values of all coefficients of the error vector:

$$TotalError=\sum_{i=1}^{k}\sum_{j=0}^{n-1}\left|e_{i}[j]\right|$$

If TotalError>L, the function returns False, meaning the sample is rejected and must be regenerated; otherwise, it returns True. The procedure is shown in Algorithm 3.
**Algorithm 3.** Error Checking Function1.$Input:\;error\;vector\;e=(e_{1},\;e_{2},\;...,\;e_{n})$2.$Compute\;TotalError=\sum_{i=1}^{k}\sum_{j=0}^{n-1}\left|e_{i}[j]\right|$3.if  TotalError>L return False else return True

This mechanism is integrated into both Kyber and Saber during the key generation and decapsulation phases, thereby improving the robustness, correctness, and security of the overall system.

6. Lightweight and Parallel Optimization Design

To enhance the deployability and responsiveness of the protocol on embedded platforms in vehicular networks, the following optimization strategies are introduced during key generation, encapsulation, and decapsulation processes:

Kyber Optimization Path

Introduce Number Theoretic Transform (NTT) to accelerate polynomial modular multiplication and convolution operations;

Significantly reduce key encapsulation and decapsulation times, improving overall protocol throughput.

Saber Optimization Path

Utilize the AVX2 instruction set to parallelize polynomial inner products and multiplications;

Vector Computation Optimization

Replace floating-point multiplications in Saber with integer approximate multiplications, reducing computational and energy overhead on embedded devices.

Sampling Function Optimization

Replace the original rejection sampling with Cumulative Distribution Table (CDT) table-based sampling, improving sampling efficiency and reducing entropy source consumption.

Error Term Limitation Mechanism

Integrate the checkE(e) process during key generation;

If the error exceeds the predefined threshold, resampling is performed to enhance protocol stability and ciphertext decapsulation success rate.

**Figure 4:**
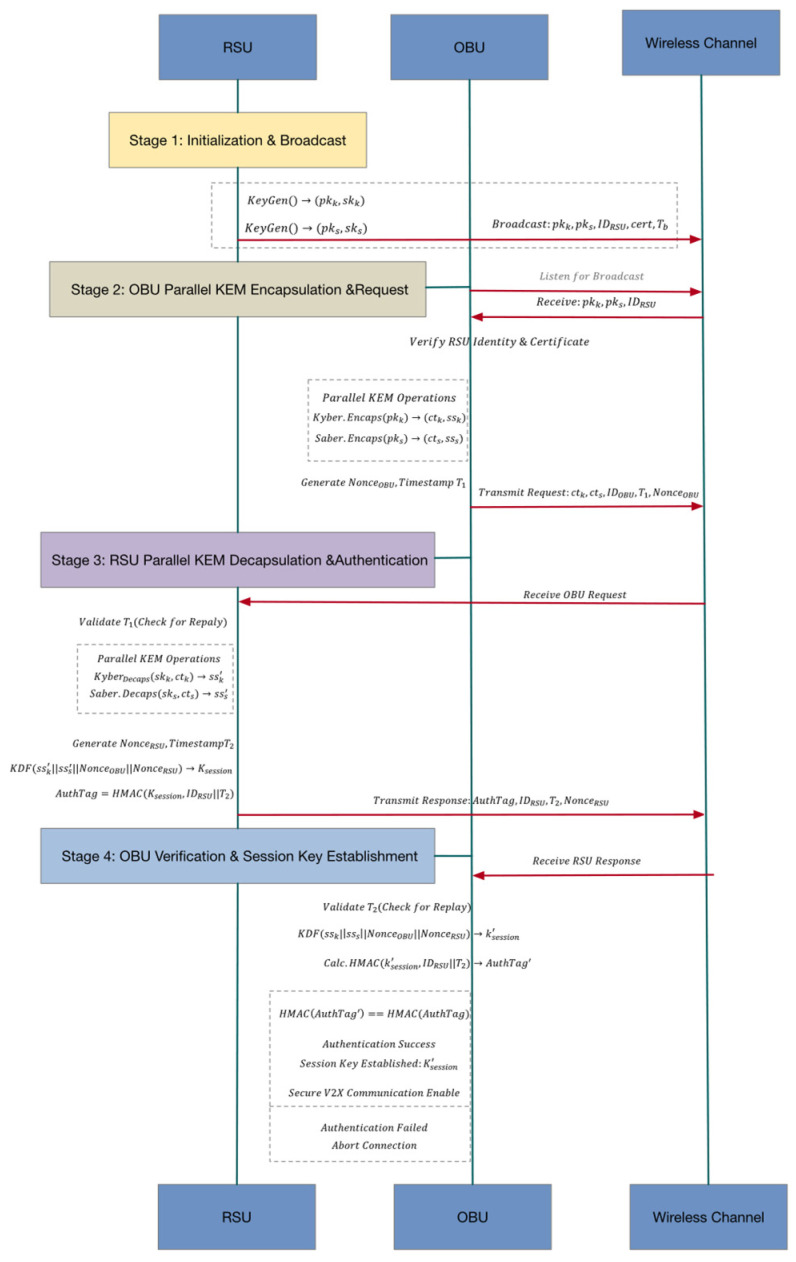
Detailed Hybrid-KEM Key Exchange Flow.

In summary, the core cryptographic primitives and interaction interfaces of the protocol have been fully defined. The complete key exchange procedure between OBU and RSU, including message sequence, parallel encapsulation/decapsulation operations, and authentication response mechanism, is illustrated in Figure 4. This figure provides an overall framework for understanding the detailed operations of the three protocol stages.

#### 3.3.2. RSU Initialization and Key Broadcast

Upon system deployment or RSU power-up, the Road-Side Unit (RSU) generates its key pairs for both Kyber and Saber and periodically broadcasts the public key information to the coverage area for OBUs (On-Board Units) to retrieve.

Key Generation Process:

$$\left({pk}_{k},{sk}_{k}\right)\leftarrow Kyber.KeyGen()$$

$$\left({pk}_{s},{sk}_{s}\right)\leftarrow Kyber.KeyGen()$$

Broadcast Contents:

Public key pair $\left({pk}_{k},{pk}_{s}\right)$

RSU identity ${ID}_{RSU}$

Protocol parameters (e.g., supported algorithm versions, configuration details)

This pre-distribution stage allows OBUs to cache public keys in advance, significantly reducing communication latency during subsequent key exchanges.

#### 3.3.3. OBU Parallel Ciphertext Encapsulation

When an OBU enters the coverage area of an RSU, it immediately retrieves the broadcast public keys and performs dual-path parallel key encapsulation.

OBU Operations:

$$\left({ct}_{k},{ss}_{k}\right)\leftarrow Kyber.Encaps({pk}_{k})$$

$$\left({ct}_{s},{ss}_{s}\right)\leftarrow Saber.Encaps({pk}_{s})$$

Handshake Message Sent:

$${Message}_{1}=\{{ct}_{k},{ct}_{s},{ID}_{OBU},T_{1}\}$$

where

${ct}_{k},{ct}_{s}$ are Kyber and Saber ciphertexts

${ID}_{OBU}$ is the OBU identity

$T_{1}$ is a timestamp to prevent replay attacks

The parallel dual encapsulation enhances both resistance to attacks and communication robustness, representing a key innovation of this protocol.

#### 3.3.4. RSU Decapsulation and Authentication Response

Upon receiving the encrypted message from the OBU, the RSU decapsulates the ciphertexts using its private keys to recover the shared key materials and derives the final symmetric session key via KDF.

RSU Decapsulation:

$${ss}_{k}^{\prime }⟵Kyber.Decaps({sk}_{k},{ct}_{k})$$

$${ss}_{s}^{\prime }⟵Saber.Decaps({sk}_{s},{ct}_{s})$$

Session Key Derivation:

$$K_{final}=KDF({ss}_{k}^{\prime }||{ss}_{s}^{\prime }),$$

Authentication Response Message:

$$MAC=HMAC(K_{final},{ID}_{RSU}||T_{2}),$$

$${Message}_{2}=\{MAC,{ID}_{RSU},T_{2}\}$$

#### 3.3.5. OBU Verification and Completion

The OBU computes the session key using its locally saved encapsulated keys:

$$K_{final}^{\prime }=KDF({ss}_{k}||{ss}_{s})$$

It then verifies the MAC received from the RSU. Upon successful verification, identity authentication is confirmed, and the session key synchronization is completed.

This stage employs a unidirectional authentication response mechanism, eliminating the additional round-trip of traditional bidirectional handshakes and improving protocol efficiency and practicality in short-lived vehicle connections.

### 3.4. Key Derivation and Authentication Mechanism

This section elaborates on the two core cryptographic components of the protocol: the Key Derivation Function (KDF) and the Hash-based Message Authentication Code (HMAC). Together, they ensure key synchronization, message authentication, and content integrity, forming the foundation of the protocol’s overall security. In this protocol, HKDF [38,39] is used as the KDF, combined with HMAC [40] for authentication and integrity verification.

#### 3.4.1. Key Derivation Function

Function and Purpose

The KDF is responsible for consolidating multiple shared key materials into a high-entropy, unique, and secure session key. In this protocol, its primary roles are:

Combining the shared keys generated by Kyber (${ss}_{k}$) and Saber (${ss}_{s}$);

Integrating random nonces generated by both communicating parties;

Producing a unified symmetric session key $k_{final}$ for HMAC authentication and encrypted communication.

This approach effectively increases key entropy and prevents security degradation in the event of a single algorithm being compromised.

Inputs

The KDF receives the following parameters:

${ss}_{k}$: Shared key output from Kyber;

${ss}_{s}$: Shared key output from Saber;

${Nonce}_{OBU}$: Random number generated by the OBU;

${Nonce}_{RSU}$: Random number generated by the RSU.

Mathematical Definition

$$k_{final}=Hash({ss}_{k}|\left|{ss}_{s}\right||{Nonce}_{OBU}||{Nonce}_{RSU}),$$

where

|| denotes bit-level concatenation;

Hash( ) denotes a cryptographic hash function, preferably SHA-256 or HKDF.

Recommended Usage:

SHA-256: outputs a 256-bit symmetric key;

HKDF: supports hierarchical key derivation and context binding for complex applications.

Security Features:

Multi-source entropy: Even if one algorithm is attacked, the combined output maintains overall session key security.

Replay and collision resistance: Nonces ensure uniqueness for each key exchange round, preventing replay attacks.

Forward secrecy: Keys are independently derived in each session, protecting past communications from compromise.

#### 3.4.2. Message Authentication Code

To ensure message integrity and authenticate its origin, the protocol employs HMAC. This mechanism binds the session key $k_{final}$ to the message content, effectively defending against forgery, man-in-the-middle attacks, and replay attacks.

HMAC Construction:

$$HMAC\left(K,m\right)=H((K⊕opad)||H((K⊕ipad)|\left|m\right))$$

,

where

K is the session key derived from the KDF ($K_{final}$);

m is the message content to authenticate (e.g., ${ID}_{RSU}||T_{2}$);

H is a hash function (recommend SHA-256);

opad: outer padding constant (0x5C repeated);

ipad: inner padding constant0x36repeated);

||: concatenation operation;

⊕: bitwise XOR.

Protocol Usage:

During Stage 3 of the handshake, the RSU computes:

$$MAC=HMAC(K_{final},{ID}_{RSU}||T_{2})$$

The RSU then sends:

$${Message}_{2}=\{MAC,{ID}_{RSU},T_{2}\}$$

The OBU uses its locally derived $K_{final}^{\prime }$ to verify the HMAC, ensuring the message is untampered and originates from a legitimate RSU.

**Figure 5:**
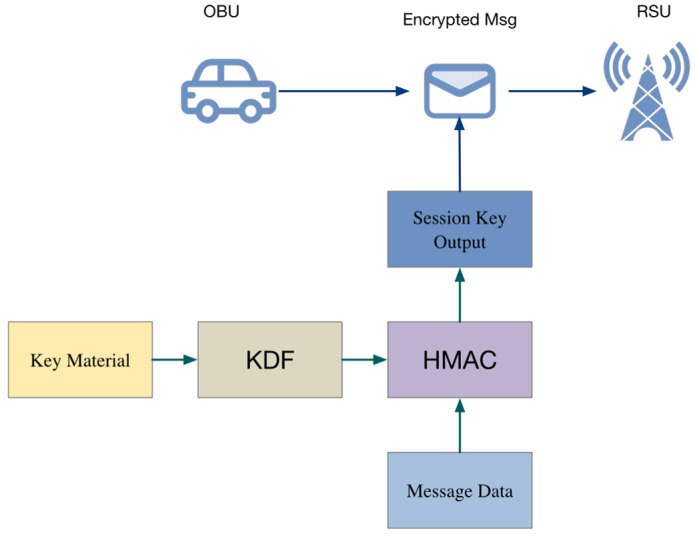
Session Key Derivation and Authentication Process Based on KDF and HMAC.

Figure 5 illustrates the interaction between the two core security components of the protocol: the KDF-based session key derivation module and the HMAC-based message authentication module.

KDF Module:

Receives the shared key materials obtained from Kyber and Saber decapsulation (e.g., ${ss}_{k},\;\;{ss}_{s}$);

Combines them with nonces exchanged during the handshake (${Nonce}_{OBU}$, ${Nonce}_{RSU}$);

Derives the final session key $K_{final}$.

HMAC Module (right, yellow block):

Uses the derived session key $K_{final}$ to compute a hash-based authentication code over the message content (e.g., ${ID}_{RSU}|\left|T_{2}\right)$);

Produces the MAC that is transmitted along with the encrypted message to the OBU or returned in the authentication response, forming a complete authentication mechanism.

OBU Verification Path (bottom):

The OBU re-computes the HMAC using its locally derived $K_{final}^{\prime }$ over the same message content.

Compares the computed HMAC with the received MAC to verify both the RSU’s authenticity and message integrity.

### 3.5. Security Properties and Comparative Evaluation

To evaluate the security and practical applicability of the proposed Kyber + Saber hybrid KEM protocol in vehicular networks (V2X), this section analyzes the protocol against typical attack models and cryptographic theory. Its protection capabilities are systematically assessed across multiple security objectives and compared with traditional key agreement schemes such as RSA and ECDH, highlighting its advantages in post-quantum security, authentication mechanisms, and key security.

#### 3.5.1. Post-Quantum Security

The core cryptographic primitives of the protocol are lattice-based:

Kyber is based on the Learning With Errors (LWE) problem.

Saber relies on the Learning With Rounding (LWR) problem.

Both problems are proven to be intractable against polynomial-time quantum attacks (e.g., Shor’s algorithm) and are recommended by NIST as post-quantum cryptographic schemes with IND-CCA2 security.

If a quantum algorithm could derive the shared key $K_{final}$ from the ciphertext pair ${(ct}_{k},{ct}_{s})$, it would imply breaking at least one of the underlying KEM primitives’ IND-CCA2 security, which is contradictory.

Thus, the protocol offers theoretical quantum resistance, maintaining key agreement security even under future quantum threats. Existing studies indicate that Kyber also demonstrates strong security in extended properties such as anonymity [41].

Resistance to Man-in-the-Middle (MitM) Attacks

The protocol uses dual-path key encapsulation combined with HMAC-based authentication, effectively mitigating MitM attacks:

Handshake process:

OBU sends the ciphertext pair:

$$\left({ct}_{t},{ct}_{s}\right)=({Enc}_{{pk}_{RSU}^{k}({ss}_{k})},{Enc}_{{pk}_{RSU}^{s}}({ss}_{s}))$$

RSU decapsulates to obtain shared secrets:

$$\left({ss}_{k},{ss}_{s}\right)=({Dec}_{{sk}_{RSU}^{k}({ct}_{k})},{Dec}_{{sk}_{RSU}^{s}}({ct}_{s}))$$

Final session key derivation:

$$k_{final}=KDF({ss}_{k}|\left|{ss}_{s}\right||{Nonce}_{OBU}||{Nonce}_{RSU})$$

MAC construction:

$MAC=HMAC(k_{final},{ID}_{RSU}||T_{2})$ An attacker cannot forge a valid MAC without $k_{final}$, which depends on dual KEM decapsulation—making forgery practically impossible.

Replay Attack Resistance

To prevent replay attacks, each communication round embeds timestamps:

OBU messages include $T_{1}$, RSU responses include $T_{2}$

The receiver verifies:

$$\left|T_{recv}-T_{current}\right|<\delta_{t}$$

If the difference exceeds the threshold ($\delta_{t}=1$), the message is rejected.

Binding the timestamp into the MAC further enhances uniqueness, preventing delayed replay.

Authentication and Message Integrity

The protocol uses lightweight HMAC for authentication, binding the identity and timing information:

$$MAC=HMAC(k_{final},{ID}_{RSU}||T_{2})$$

Verification at OBU:

$${MAC}_{recv}=HMAC(k_{final}^{\prime },{ID}_{RSU}||T_{2})$$

Matching MACs confirm both the RSU’s authenticity and message integrity. HMAC’s collision resistance and key sensitivity prevent attackers from forging MACs without $k_{final}$.

Key Uniqueness and Forward Secrecy

To prevent key reuse and protect past sessions, the protocol introduces dual nonces for each round:

$$k_{final}^{i}=KDF({ss}_{k}^{i}||{ss}_{s}^{i}||{Nonce}_{OBU}^{i}||{Nonce}_{RSU}^{i})$$

As long as at least one nonce differs between rounds:

$$k_{final}^{i}\neq k_{final}^{j}$$

Even if a past session key is compromised, future keys remain secure, ensuring forward secrecy and key isolation.

In addition to resisting quantum attacks, the proposed protocol maintains strong defense against classical network threats. A timestamp-based HMAC verification scheme ensures message freshness and integrity, effectively preventing replay and injection attacks. During key negotiation, a dual-path KEM structure allows both the OBU and RSU to generate independent random values and session keys, so any tampering or ciphertext replacement immediately causes validation failure, blocking man-in-the-middle attempts. In the key-derivation phase, the combination of KDF and HMAC binding produces unique and unpredictable session keys, making reconstruction of master secrets from intercepted packets infeasible. These mechanisms collectively preserve confidentiality and authentication integrity under hybrid classical–quantum attack models. Overall, the hybrid Kyber + Saber key agreement protocol provides post-quantum security together with multi-layer protection against conventional network threats in practical vehicular environments.

#### 3.5.2. Resistance to Jamming and DoS Attacks

Beyond cryptographic protection and authentication, the proposed protocol also exhibits inherent resilience against jamming and denial-of-service (DoS) attacks at the communication layer. Traditional multi-round handshake protocols require multiple message exchanges, leading to long communication durations and high channel occupancy; under continuous interference, these handshake processes are easily disrupted. In contrast, the proposed Kyber + Saber hybrid key agreement mechanism completes the entire core key exchange with only one request and one response, keeping the overall handshake delay within the millisecond range and significantly reducing the exposure window to interference.

In addition, a key caching and fast reconnection mechanism is introduced during the session maintenance phase. When vehicle movement or channel interruption causes a temporary session failure, the system can re-establish the connection based on the most recent valid key state without executing the full handshake again. This improves connection robustness under highly dynamic network conditions.

Simulation results demonstrate that even under interference and congestion conditions, the proposed protocol maintains a handshake success rate (HSR) above 98%, verifying its robustness against disturbances at both the physical and transport layers. Consistent with previous findings [42], reported that reducing handshake rounds and employing lightweight key caching mechanisms can significantly enhance communication success rates and resilience in V2X systems under interference and blockage scenarios. The results of this study further validate that conclusion.

Therefore, the proposed Kyber + Saber lightweight key agreement protocol not only resists logical-layer attacks such as replay and man-in-the-middle attacks, but also demonstrates strong anti-jamming and self-recovery capabilities in real vehicular communication environments, making it well-suited for diverse and complex vehicular network scenarios.

#### 3.5.3. Security Reduction and Theoretical Proof

The overall security of the proposed protocol can be theoretically analyzed from two perspectives. First, we adopt a Game Hopping approach to reduce the adversary’s advantage in successfully attacking the protocol to the hardness of breaking the underlying Kyber and Saber algorithms, thereby formally proving its IND-CCA2 security. Second, we analyze the security of the authentication component.

(1) Game-Hopping-Based Proof of IND-CCA2 Security

To formally demonstrate that the protocol achieves security in the IND-CCA2 model, we employ the Game Hopping technique. By constructing a sequence of games ($G_{0}\to G_{4}$), we gradually replace critical elements of the protocol and analyze the adversary’s distinguishing advantage in each step. The reduction shows that any non-negligible advantage in breaking the protocol implies the ability to break Kyber, Saber, or the KDF. The detailed structure of the game-hopping sequence is summarized in Table 1.

Explanations:

Game ($G_{0}$): Represents the real execution of the protocol, where the adversary interacts with the system and attempts to distinguish the real session key from a random key.

$${Adv}_{A}^{G_{0}}=|\mathrm{Pr}\left[S_{0}\right]-\frac{1}{2}|$$

Game ($G_{1}$): The ciphertext and shared secret generated by Saber are replaced with uniformly random values. By the IND-CPA security of Saber (which is the basis for its IND-CCA2 security), the adversary cannot distinguish ($G_{0}$) from ($G_{1}$). Thus,

$$\left|\mathrm{Pr}\left[S_{1}\right]-Pr[S_{0}]|\leq {Adv}_{Saber}^{IND-CPA}(A\right)$$

Game ($G_{2}$): The ciphertext and shared secret generated by Kyber are also replaced with random values. By the IND-CPA security of Kyber, the adversary cannot distinguish ($G_{1}$) from ($G_{2}$). Therefore,

$$\left|\mathrm{Pr}\left[S_{2}\right]-Pr[S_{1}]|\leq {Adv}_{kyber}^{IND-CPA}(A\right)$$

At this point, the dual-path KEM outputs have been fully randomized.

Game ($G_{3}$): All critical parameters are now randomized. Since the KDF inputs $\left({ss}_{k},{ss}_{s}\right)$ are independent high-entropy random values, the pseudorandomness of the KDF ensures that its output is computationally indistinguishable from a truly random value. Thus,

$$\left|\mathrm{Pr}\left[S_{3}\right]-Pr[S_{2}]|\leq {Adv}_{KDF}^{IND-CPA}(A\right)$$

Game ($G_{4}$): In the final game, the challenger always returns the same output, and the adversary has no distinguishing advantage:

$$\mathrm{Pr}\left[S_{3}\right]=Pr[S_{4}]$$

Security Conclusion:

By combining the above transitions, the adversary’s advantage in the real protocol execution is strictly bounded as:

$${Adv}_{A}^{IND-CCA2}\left(\lambda \right)=\left|\mathrm{Pr}\left[S_{0}\right]-\frac{1}{2}\right|\leq {Adv}_{Kyber}^{IND-CPA}+{Adv}_{Saber}^{IND-CPA}+{Adv}_{KDF}^{IND-CPA}+negl(\lambda )$$

Since Kyber and Saber have been proven to be IND-CPA secure under their respective hardness assumptions, and the KDF is modeled as a pseudorandom function, all terms are negligible. Therefore, the adversary’s advantage in breaking the proposed hybrid protocol is also negligible. This provides a rigorous theoretical foundation for the IND-CCA2 security of the scheme and complements the earlier analysis of its resistance against quantum adversaries.

(2) Unforgeability of HMAC

The identity authentication and message integrity of the protocol rely on the security of HMAC. Suppose an adversary attempts to forge an authentication code without knowledge of the key $K_{final}$:

$${MAC}^{*}=HMAC\left(k_{final},m^{*}\right)\;\;for\;some\;m^{*}\neq m$$

Under the standard HMAC security model, the probability of successful forgery by the adversary is upper-bounded by:

$$\mathrm{Pr}\left[Forge\right]\leq \frac{1}{2^{\lambda }}+{Adv}_{HMAC}^{EUF-CMA}(A)$$

where λ is the output bit length, and ${Adv}_{HMAC}^{EUF-CMA}$ represents the advantage of the adversary in existential forgery under chosen-message attacks. Since $K_{final}$ can only be derived by legitimate parties through the secure KEM and KDF process described above, the adversary cannot obtain a valid key and therefore cannot generate a legitimate MAC value without querying.

The final receiver performs verification:

$$Verify:HMAC(K_{final}^{\prime },{ID}_{RSU}||T_{2})={MAC}_{received}$$

If they do not match, the message is immediately discarded. This ensures the resistance against man-in-the-middle attacks and replay attacks described earlier.

(3) Comprehensive Reduction

$${Adv}_{A}^{total}\leq {Adv}_{Kyber}^{IND-CPA}+{Adv}_{Saber}^{IND-CPA}+{Adv}_{KDF}^{IND-CPA}+{Adv}_{HMAc}^{EUF-CMA}+negl(\lambda )$$

All advantage terms are based on well-accepted hardness problems or the security of standard cryptographic primitives. Therefore, the overall advantage of the adversary is negligible. Furthermore, although this paper has completed the theoretical game hopping proof, future work could consider using automated formal verification tools (such as ProVerif) to perform more comprehensive modeling and verification of the protocol’s authentication logic, communication timing, and other properties, thereby further enhancing the credibility of the protocol’s security.

Through a structural design combining dual-path KEM, HMAC-based strong authentication, timestamp binding, and Nonce-enhanced entropy sources, the proposed protocol demonstrates distinct advantages across several critical security attributes. Table 2 summarizes the comparative security objectives of the Kyber + Saber hybrid KEM protocol against conventional RSA and ECDH protocols in V2X environments.

(4) Formal Security Model and Verification Framework

In addition to the above game-hopping reduction, the proposed scheme can also be formally described under the Bellare–Rogaway authenticated key-exchange framework. Within this model, the adversary is granted full control over the communication channel, including the ability to intercept, replay, or modify any message. All system parameters are assumed to be authenticated, while the employed hash and key derivation functions are modeled as random oracles. Under these assumptions, the protocol satisfies key indistinguishability and forward secrecy: if an adversary could distinguish the established session key from a random value or compromise its freshness, it would directly imply breaking the IND-CCA2 security of Kyber or Saber, or the pseudorandomness of the KDF, all of which are proven to be negligible under standard hardness assumptions. The message authentication and integrity are likewise ensured by the existential unforgeability of HMAC under chosen-message attacks. Future work will further include automated formal verification using tools such as ProVerif or AVISPA to simulate adversarial interactions and validate authentication logic and timing consistency, thereby enhancing the completeness of the formal proof in both theoretical and simulation-based contexts.

#### 3.5.4. Comparative Analysis with Other PQC Algorithms

In addition to classical schemes such as RSA and ECDH, the proposed hybrid Kyber + Saber protocol can also be conceptually compared with other post-quantum cryptographic (PQC) algorithms recommended by NIST, including FrodoKEM, NTRU, and BIKE. These algorithms are based on three distinct hardness assumptions—standard lattices, ideal lattices, and code-based cryptography—each presenting unique trade-offs among computational efficiency, key size, and implementation complexity.

FrodoKEM, derived from the standard Learning With Errors (LWE) problem, offers strong theoretical resistance to quantum attacks but involves relatively large key sizes and high computational costs, which limit its applicability to resource-constrained vehicular devices such as On-Board Units (OBUs) and Road-Side Units (RSUs). NTRU, constructed from the hardness of ideal lattices, features smaller keys and faster performance; however, its decryption process may exhibit instability against adaptive attacks if parameter selection is not handled carefully. BIKE, designed on code-based cryptography using quasi-cyclic codes, provides compact keys and solid security, yet relies on iterative bit-flipping decoding, which may introduce higher and less predictable latency—undesirable in real-time vehicular scenarios that demand deterministic communication.

By contrast, the proposed Kyber + Saber hybrid design integrates the Module-LWE and Module-LWR assumptions, combining Kyber’s strong theoretical security and NTT-based acceleration with Saber’s integer-only arithmetic and power-efficient computation. This dual-path encapsulation mechanism enhances key freshness and handshake reliability while maintaining lightweight message structures and low bandwidth consumption.

From a theoretical perspective, the hybrid Kyber + Saber protocol is expected to achieve lower computational overhead than FrodoKEM, comparable or slightly better handshake latency than NTRU, and more stable real-time behavior than BIKE in dynamic vehicular environments. These expectations stem from the protocol’s structural characteristics—its dual encapsulation path, reduced arithmetic complexity, and balanced cryptographic workflow—which collectively improve computational efficiency and communication stability.

Overall, the proposed hybrid scheme achieves a favorable balance between post-quantum security, communication efficiency, and implementation feasibility, serving as a practical and scalable reference model for secure vehicular communications in the forthcoming quantum era.

#### 3.5.5. Summary of Hybrid KEM Protocol

The proposed hybrid KEM protocol demonstrates:

Enhanced security: Combining Kyber and Saber provides structural redundancy and fault tolerance—compromise of one primitive does not jeopardize overall security.

Reliable authentication: Lightweight HMAC plus timestamp ensures message authenticity and integrity without significant communication overhead.

V2X suitability: Optimized for low-latency, resource-constrained vehicular networks, supporting efficient key updates and secure communications under quantum threats.

Overall, the protocol offers strong post-quantum security, robust authentication, and high practical efficiency, making it well-suited for future vehicular network applications.

## 4. Performance Evaluation and Comparative Analysis

To evaluate the application performance of the proposed Kyber + Saber hybrid Key Encapsulation Mechanism (KEM) protocol in vehicular networks (V2X), this chapter designs and implements simulation experiments. The focus is on verifying whether the protocol can maintain post-quantum security, low latency, and controllable system overhead in highly dynamic, resource-constrained, and security-sensitive vehicular networks. The simulation platform is based on Veins + SUMO, and the performance metrics include communication delay, key generation time, CPU/memory usage, and data throughput.

### 4.1. Simulation Platform and Test Plan

#### 4.1.1. Simulation Platform Configuration

This section describes the hardware and software environment, simulation scenario setup, protocol groups, and evaluation metrics, providing a foundation for the subsequent performance evaluation and comparative analysis. The experiments were conducted on a virtual machine with instant-veins-5.2-i1 installed, which offers a ready-to-use vehicular network simulation framework integrating OMNeT++ 5.1 for network simulation and SUMO 1.8.0 for traffic modeling, including multiple runnable typical scenarios. The virtual machine hardware and system configuration are summarized in Table 3.

All simulation tools and software used in this study are identified with their version numbers and developers: OMNeT++ 5.1 (OpenSim Ltd., Budapest, Hungary), SUMO 1.8.0 (DLR—German Aerospace Center, Berlin, Germany), and Debian 11 (Debian Project, SPI Inc., New York, NY, USA). The experiments integrate Kyber (LWE-based) and Saber (LWR-based) algorithm modules into the Veins communication stack, replacing the default security module. Comparative groups include RSA and ECDH, which are implemented under the same software environment to ensure fairness. This configuration enables a comprehensive assessment of end-to-end performance, resource consumption, and security features of different key exchange protocols under realistic vehicular network conditions.

The experiments integrated Kyber (LWE-based) and Saber (LWR-based) algorithm modules into the Veins communication stack, replacing the default security module. Comparative groups included RSA and ECDH, which were implemented under the same software environment to ensure fairness. The Kyber512 and LightSaber implementations were optimized based on the PQClean project, leveraging Number Theoretic Transform (NTT) for Kyber and integer approximation multiplication for Saber, combined with AVX2 instructions to accelerate computation. Custom protocol modules were integrated into the OBU and RSU application layers, supporting message encryption and key agreement. Data collection was performed using OMNeT++ scalar and vector recording for delay, CPU, and memory usage, while the TraCI interface was used to interact with SUMO to obtain vehicle positions, speeds, and traffic flow status. The simulation modules were based on TraCIDemo11p/TraCIDemoRSU11p, with added Kyber + Saber encapsulation and decapsulation functions. The implementation details of all key exchange protocols are summarized in Table 4.

This configuration enables a comprehensive assessment of the end-to-end performance, resource consumption, and security features of different key exchange protocols under realistic vehicular network conditions.

Limitation Statement: It should be noted that the experiments in this study were conducted in a virtualized environment. The measured end-to-end latency and resource overhead include additional overhead from virtual machine scheduling, the simulation framework (OMNeT++/Veins), and background operating system processes. Thus, while the results effectively reflect the relative performance comparison and trends at the system level, there may be discrepancies compared to absolute performance data obtained on real bare-metal hardware (such as dedicated OBU/RSU devices). The primary objective of this experimental setup was to validate the effectiveness and superiority of the proposed protocol compared to traditional schemes in a controlled and fair comparative environment.

#### 4.1.2. Simulation Scenario Settings

To evaluate the protocol’s performance variations under different traffic densities, this study conducted comparative simulations with 50, 100, and 195 vehicular nodes. Among them, 50 and 100 vehicles represent sparse and medium traffic conditions, simulating typical suburban or urban road environments, while 195 vehicles follow the official Veins/SUMO benchmark configuration, corresponding to a high-density city traffic scenario.

The selection of these node scales was based on two primary considerations: (1) ensuring consistency with the open-source reference scenarios so that the simulation environment maintains comparability in terms of traffic flow and road topology; and (2) confirming that 195 vehicles can stably emulate high-load communication conditions within the Veins framework, enabling observation of performance variations and handshake success rates under dense vehicular conditions.

According to previous studies, vehicular network simulations typically employ 100–200 nodes to characterize representative high-density traffic scenarios. Therefore, the configuration used in this study is both realistic and technically representative for evaluating vehicular communication protocols.

Simulation experiments were conducted in a 2500 m × 2500 m two-dimensional urban area with a road height of 50 m. The traffic scenario represents a typical city road environment. A total of 195 vehicles (SUMO flow0) of type vtype0 were deployed, each with an acceleration of 2.6 m/s^2^, a maximum speed of 14 m/s, a length of 2.5 m, and a minimum gap of 2.5 m. Vehicles entered the simulation sequentially every 3 s, with initial speeds following a normal distribution (σ = 0.5); a fixed random seed was used to ensure reproducibility.

A single RSU was deployed at coordinates (2000, 2000, 3) m, where vehicles moved along predefined routes and established secure connections with the RSU. The communication range was set to a maximum interface distance of 2600 m, with a 1D monopole antenna configuration.

Three protocol groups were evaluated in this environment: the proposed post-quantum hybrid KEM using Kyber + Saber, traditional RSA, and ECDH. These settings allowed for a controlled comparison of protocol performance, resource consumption, and security characteristics under realistic vehicular network conditions.

**Figure 6:**
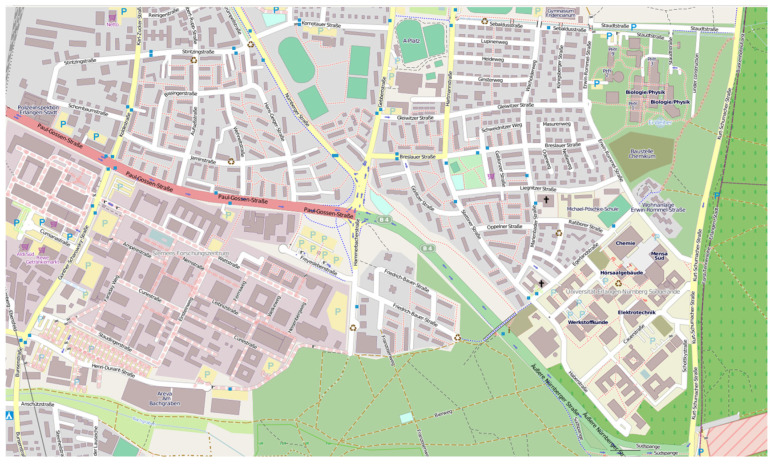
Urban road map for simulation.

Figure 6 shows the city road map used for simulation, showing the urban traffic environment.

**Figure 7:**
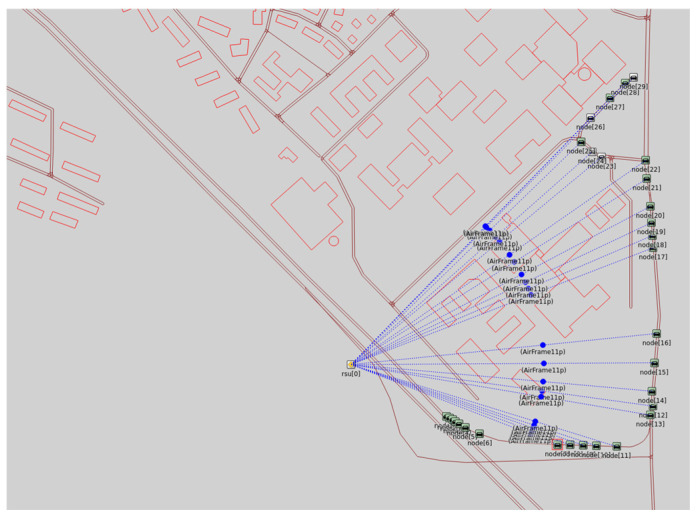
RSU–OBU node deployment and communication. Note: The bracketed labels (e.g., node[12], AirFrame[11p]) denote internal identifiers in OMNeT++/Veins, not literature references.

Figure 7 shows the node deployment and RSU–OBU communication illustration in Veins, showing vehicle movement along predefined routes and secure connections with the RSU.

#### 4.1.3. Evaluation Metrics and Methods

The performance of the proposed hybrid Kyber + Saber protocol is evaluated across four dimensions: latency, computation, resource use, and communication cost. Latency covers the key handshake delay and message authentication delay (MAD), while computation overhead includes key generation, encapsulation/decapsulation, and KDF-HMAC execution. Resource consumption is measured by CPU and memory usage, and communication cost is assessed through handshake rounds, message size, and total traffic volume. Throughput and scalability are further analyzed under varying node densities (50, 100, and 195 vehicles).

All experiments were conducted on the Veins platform, using OBUs and RSUs as test nodes. Each test was repeated at least ten times, and results were reported as mean ± standard deviation (SD) for reliability. The definitions and measurement methods of all metrics are summarized in Table 5.

The above metrics comprehensively evaluate multiple aspects of the proposed protocol’s performance in vehicular network environments. Latency metrics, including Key Handshake Delay and Message Authentication Delay (MAD), assess the responsiveness of the protocol under stringent low-latency conditions that are typical of V2X communication. Computational overhead, represented by KeyGen, Enc/Dec, and KDF/HMAC execution times, reflects the intrinsic algorithmic complexity and efficiency of cryptographic operations. Resource consumption, which includes CPU utilization and memory usage, measures the adaptability of the scheme to embedded vehicular platforms with constrained computational resources. Communication cost, characterized by the number of handshake rounds, message length, and total traffic volume, indicates the protocol’s link utilization efficiency and its impact on wireless bandwidth.

To ensure statistical reliability, each experiment was repeated at least ten times, and the results were reported as mean ± standard deviation (SD). Confidence intervals and error bars were generated for key performance indicators using MATLAB R2022b (MathWorks Inc., Natick, MA, USA) and Python 3.11 (Python Software Foundation, Wilmington, DE, USA), ensuring the statistical significance and reproducibility of all presented results.

The experimental statistical methodology is as follows: each test group was repeated no fewer than ten times, and the mean ± standard deviation was computed for each performance metric. Key indicators were plotted with 95% confidence intervals (CI = 0.95) and error bars to visualize the variation range.

To comprehensively capture the worst-case performance fluctuations, the study not only recorded the average handshake latency of successful sessions but also collected data on failed and recovered handshake samples. Random packet loss, channel blocking, and node interruption events were introduced in Veins to simulate realistic network disturbances. The Handshake Success Rate (HSR), number of retransmission attempts, and recovery delay ($T_{recover}$) were calculated, and their upper and lower CI bounds were used to evaluate protocol stability under abnormal conditions.

For statistical reliability, the 95% confidence level was adopted, and both MATLAB and Python were used to generate error-bar plots, ensuring the results’ statistical significance and reproducibility. This analysis approach not only highlights the protocol’s average performance under ideal conditions but also demonstrates its robustness and recovery capability under extreme operational scenarios, providing valuable references for hardware implementation and real-world deployment.

To emulate unstable communication conditions in vehicular networks, the simulation introduced both Failed Handshake and Recovery Scenario tests. A Failed Handshake refers to cases where message exchanges between the OBU and RSU fail to complete key synchronization due to channel congestion, packet loss, or timeout. A Recovery Scenario denotes the protocol’s ability to resume secure communication through a fast retransmission and session resumption mechanism within 1–2 round-trip times (RTT).

During data collection, the number of successful handshakes ($N_{\mathrm{success}}$), failed handshakes ($N_{\mathrm{fail}}$), and recovered sessions ($N_{\mathrm{rec}}$) were recorded. The Handshake Success Rate (HSR) and Recovery Success Rate ($R_{\mathrm{rec}}$) were then defined as follows:

$$HSR=\frac{N_{\mathrm{success}}}{N_{\mathrm{success}}+N_{\mathrm{fail}}}\times 100\%,R_{\mathrm{rec}}=\frac{N_{\mathrm{rec}}}{N_{\mathrm{fail}}}\times 100\%.$$

Confidence interval upper bounds represent the worst-case latency (including failure and recovery samples), while lower bounds correspond to optimal successful handshake performance.

### 4.2. Protocol Complexity and Resource Adaptability Analysis

To comprehensively evaluate the applicability of the proposed lightweight key agreement protocol based on Kyber and Saber in vehicular networks, this section compares the performance of Kyber + Saber, ECDH, and RSA across three dimensions: communication complexity, computational complexity, and resource adaptability.

#### 4.2.1. Communication and Computation Complexity Analysis

In vehicular key agreement, communication complexity is a critical metric for evaluating protocol practicality, particularly in high-dynamic and low-latency V2X environments. Key factors include the number of message rounds during the handshake, the size of each transmitted message, and the total communication load. These parameters collectively determine the protocol’s impact on wireless bandwidth, transmission latency, and system resource scheduling. To provide a clear illustration of communication overhead, Table 6 compares the proposed Kyber + Saber protocol with mainstream ECDH and RSA protocols across these three metrics.

In vehicular key agreement, communication complexity depends on the number of message exchanges, packet size, and total data volume, which directly affect bandwidth and latency. As shown in Table 6, the Kyber + Saber hybrid KEM introduces about 5.2 KB of handshake overhead—slightly higher than classical schemes—because of its dual-path encapsulation transmitting two ciphertexts and authentication data. However, it completes authentication and key agreement within only 1.5 rounds, compared with 2 rounds for RSA and ECDH, reducing air-interface usage and delay.

Although its packet size (≈2.6 KB) exceeds that of ECDH (0.5 KB) and RSA (1.2 KB), the single-shot parallel transmission design improves link efficiency and shortens negotiation time. Fixed-length encoding and optimized Nonce, Timestamp, and HMAC fields also enable direct parsing and hardware-level acceleration, minimizing context-switch and serialization overhead. Overall, the Kyber + Saber protocol achieves fewer handshake rounds, low latency, and strong robustness, making it well-suited for high-mobility, short-session V2X environments.

#### 4.2.2. Computation Complexity and Latency Estimation

**Figure 8:**
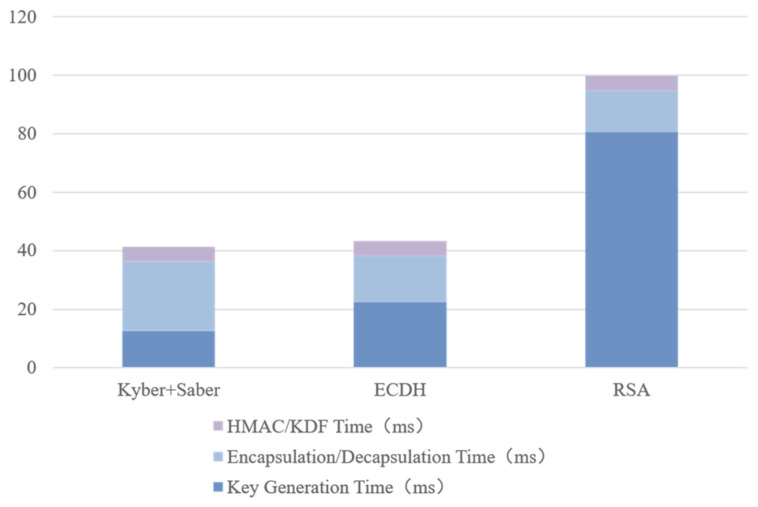
Computation Delay Distribution Across Protocol Stages in Different Key Agreement Schemes.

To evaluate the end-to-end latency distribution and computational overhead of the three protocols, stage-wise measurements were performed in the Veins simulation environment, recording the average execution times of Key Generation (KeyGen), Encapsulation/Decapsulation (Encaps/Decaps), and Session-Key Derivation and Authentication (KDF/HMAC). The experimental results are summarized in Table 7 and Table 8, and visually illustrated in Figure 8.

As shown in Figure 8, the RSA protocol exhibits the highest latency (~98.9 ms), with over 80% spent on key generation due to large-integer modular operations and certificate validation overhead. ECDH performs faster (41.6 ms) but remains limited by scalar multiplication and two-round handshakes, which reduce responsiveness.

The proposed Kyber + Saber hybrid protocol achieves a total delay of 41.2 ms, including 12.6 ms for key generation, 23.7 ms for encapsulation/decapsulation, and 4.9 ms for KDF/HMAC. Although its latency is similar to ECDH, it offers stronger post-quantum security and better scalability. The reduction mainly stems from NTT-based modular multiplication in Kyber, AVX2 vector parallelization in Saber, and integer inner-product approximations that lower CPU load and energy use.

All latency values include I/O, serialization, and 802.11p link modeling, thus reflecting realistic end-to-end performance. Overall, Kyber + Saber achieves comparable delay to ECDH with higher security and lower computational cost, confirming its suitability for low-latency, quantum-resilient V2X applications.

#### 4.2.3. Resource Adaptability and Deployment Cost

Based on Table 8, this section evaluates the computational adaptability and deployment cost of RSA, ECDH, and the proposed Kyber + Saber protocol in vehicular networks. CPU utilization and memory consumption serve as key indicators of feasibility on resource-constrained OBUs and RSUs.

The Kyber + Saber protocol demonstrates the best efficiency, with average CPU usage of 45.3% and memory consumption of 34.68 MB—lower than ECDH (56.8%, 39.11 MB) and RSA (72.5%, 68.12 MB). This improvement results from its hybrid-KEM structure: Kyber accelerates modular polynomial operations via NTT, while Saber applies AVX2-based integer inner products to reduce floating-point cost and cache load. Its single-round handshake with one-way authentication further eliminates redundant computation.

In contrast, RSA shows the highest resource demand, making it unsuitable for embedded or edge deployment. ECDH performs moderately but still incurs notable key-generation overhead, consistent with TLS studies highlighting high energy cost in constrained devices [43]. Recent research on embedded PQC and Kyber hardware acceleration [44,45] further supports the feasibility of lattice-based protocols for vehicular applications.

Overall, Kyber + Saber achieves secure post-quantum communication with minimal resource consumption, offering excellent adaptability for low-latency and energy-efficient V2X systems.

### 4.3. Performance Summary

Based on the end-to-end comparison of the proposed Kyber + Saber hybrid KEM with RSA and ECDH in V2X environments, several key conclusions can be drawn. The hybrid scheme achieves superior performance–security balance, with lower handshake latency, faster key generation, and reduced resource consumption, making it ideal for deployment on resource-constrained OBUs and RSUs. The reported results reflect complete end-to-end measurements—including cryptographic execution, Veins simulation, and system overhead—thus providing realistic estimations of practical deployment performance.

Although RSA and ECDH remain effective under classical assumptions, their reliance on integer factorization and elliptic-curve discrete logarithms exposes them to quantum vulnerabilities. Hence, they may fail to satisfy future post-quantum V2X security requirements.

Given the high mobility and short connection lifetimes of vehicular networks, the Kyber + Saber protocol offers clear practical advantages in latency, adaptability, and robustness, as summarized in Table 9.

In summary, the Kyber + Saber hybrid protocol provides strong security while maintaining efficient end-to-end resource utilization and operational performance. Its low handshake latency, moderate resource consumption, and high compatibility with embedded platforms make it a highly feasible candidate for engineering deployment, positioning it as a promising solution for next-generation V2X communications.

## 5. Conclusions and Future Work

This study proposes a hybrid key encapsulation mechanism (Hybrid-KEM) protocol for vehicular networks (V2X), integrating the post-quantum algorithms Kyber and Saber to achieve balanced security, latency, and resource adaptability. Using parallel KEM encapsulation, HMAC-based verification, and a one-round handshake, the protocol ensures quantum-resistant and efficient communication while resisting replay and man-in-the-middle attacks.

Experimental results on the Veins + SUMO platform demonstrate that Kyber + Saber achieves lower handshake delay, faster key generation, and reduced resource consumption compared with RSA and ECDH, proving its suitability for dynamic, resource-constrained vehicular environments. The protocol’s NTT and AVX2 optimizations, along with integer-based inner products, enhance efficiency and hardware compatibility on OBUs and RSUs.

Future work will focus on improving scalability through multithreading, batch authentication, and cooperative edge computing, as well as extending the framework to adaptive hybrid PQC schemes such as FrodoKEM and NTRU. Furthermore, to address scalability and network-level performance evaluation, future work will extend the current experimental setup beyond single OBU–RSU interactions to multi-node vehicular environments. Large-scale simulations integrating Veins and SUMO, or alternative frameworks such as NS-3, will be designed to model dynamic road topologies involving multiple vehicles and RSUs operating concurrently. This will enable a deeper analysis of the protocol’s scalability, handshake concurrency, and message reliability under varying vehicular densities and mobility patterns. Through such network-scale evaluations, the proposed hybrid KEM scheme can be further validated for deployment in realistic 5G-V2X environments where multiple nodes perform simultaneous secure handshakes. Real-world validation on Raspberry Pi and ARM Cortex platforms integrated with 5G/C-V2X stacks will be pursued. Additionally, countermeasures against side-channel and hybrid quantum attacks—such as formal verification, device binding, and dynamic key updates—will further strengthen practical security.

Beyond the theoretical security challenges introduced by quantum computing, post-quantum cryptographic algorithms also face practical risks from side-channel attacks (SCA) in hardware implementations. Such attacks exploit power consumption, timing variations, or electromagnetic emissions to infer secret keys, threatening the protocol’s security at the physical layer. Although this study focuses on protocol-level design and performance optimization, future work will address the implementation security of Kyber and Saber on embedded platforms. Key directions include adopting constant-time computation to eliminate data-dependent timing leakage, applying masking and noise-injection techniques during polynomial multiplication and key generation to counter power analysis, and integrating Hardware Security Modules (HSMs) to isolate critical key operations within trusted hardware. In addition, a comprehensive simulation and evaluation framework based on power, timing, and fault-injection models will be developed to verify resistance against physical-layer threats. These efforts will further enhance the robustness and long-term reliability of the Kyber + Saber hybrid protocol in real vehicular communication deployments.

Overall, the Kyber + Saber hybrid KEM protocol proposed in this study offers a feasible path toward post-quantum security, end-to-end low resource consumption, and embedded deployment adaptability, providing an important reference for the communication security of intelligent transportation systems in the future quantum era. Follow-up work will continue to optimize the protocol structure, expand algorithm adaptability and engineering deployment capabilities, and promote its standardization and practical implementation.

## Figures and Tables

**Table 1:** Game-Hopping Sequence for IND-CCA2 Proof.

Security Games			
$Game\;G_{0}$:		$Game\;G_{1}$:	
1.	$\left({pk}_{k},{sk}_{k}\right)\leftarrow Kyber.KeyGen()$	1.	$\left({pk}_{k},{sk}_{k}\right)\leftarrow Kyber.KeyGen()$
2.	$\left({pk}_{s},{sk}_{s}\right)\leftarrow Saber.KeyGen()$	2.	$\left({pk}_{s},{sk}_{s}\right)\leftarrow Saber.KeyGen()$
3.	$\left({ct}_{k},{ss}_{k})\leftarrow Kyber.Encaps({pk}_{k}\right)$	3.	(${ct}_{k},{ss}_{k})\leftarrow Kyber.Encaps({pk}_{k})$
4.	$\left({ct}_{s},{ss}_{s}\right)\leftarrow Saber.Encaps({pk}_{s})$	4.	${ct}_{s}\leftarrow U(C_{s})$
5.	$K_{final}$ $\leftarrow KDF({ss}_{k}|\left|{ss}_{s}\right||{Nonce}_{OBU}||{Nonce}_{RSU})$	5.	${ss}_{s}\leftarrow U({SS}_{s})$
6.	$\hat{K}\leftarrow U({\left\{0,1\right\}}^{\lambda })$	6.	$K_{final}$ $\leftarrow KDF({ss}_{k}|\left|{ss}_{s}\right||{Nonce}_{OBU}||{Nonce}_{RSU})$
7.	b←U({0, 1})	7.	$\hat{K}\leftarrow U({\left\{0,\;1\right\}}^{\lambda })$
8.	if b=0: $return\;({pk}_{k},{pk}_{s},{ct}_{k},{ct}_{s},K_{final})$ else: $\;\;\;return\;({pk}_{k},{pk}_{s},{ct}_{k},{ct}_{s},\hat{K})$	8.	b←U({0, 1})
		9.	if b=0: $\;\;\;return\;({pk}_{k},{pk}_{s},{ct}_{k},{ct}_{s},K_{final}$ else: $\;\;\;return\;({pk}_{k},{pk}_{s},{ct}_{k},{ct}_{s},\hat{K})$
Game $G_{2}$**:**		Game $G_{3}$**:**	
1.	$\left({pk}_{k},{sk}_{k}\right)\leftarrow Kyber.KeyGen()$	1.	${pk}_{k}\leftarrow U({PK}_{k})$
2.	$\left({pk}_{s},{sk}_{s}\right)\leftarrow Saber.KeyGen()$	2.	${pk}_{s}\leftarrow U({PK}_{s})$
3.	${ct}_{k}\leftarrow U(k)$	3.	${ct}_{k}\leftarrow U(k)$
4.	${ss}_{k}\leftarrow U({SS}_{k})$	4.	${ss}_{k}\leftarrow U({SS}_{k})$
5.	${ct}_{s}\leftarrow U(C_{s})$	5.	${ct}_{s}\leftarrow U(C_{s})$
6.	${ss}_{s}\leftarrow U({SS}_{s})$	6.	${ss}_{s}\leftarrow U({SS}_{s})$
7.	$K_{final}$ $\leftarrow KDF({ss}_{k}|\left|{ss}_{s}\right||{Nonce}_{OBU}||{Nonce}_{RSU})$	7.	$K_{final}\leftarrow U({\left\{0,\;1\right\}}^{\lambda })$
8.	$\hat{K}\leftarrow U({\left\{0,\;1\right\}}^{\lambda })$	8.	$\hat{K}\leftarrow U({\left\{0,\;1\right\}}^{\lambda })$
9.	b←U({0, 1})	9.	b←U({0, 1})
10.	if b=0: $return\;({pk}_{k},{pk}_{s},{ct}_{k},{ct}_{s},K_{final})$ else: $\;\;\;return\;({pk}_{k},{pk}_{s},{ct}_{k},{ct}_{s},\hat{K})$	10.	if b=0: $\;\;\;return\;({pk}_{k},{pk}_{s},{ct}_{k},{ct}_{s},K_{final})$ else: $\;\;\;return\;({pk}_{k},{pk}_{s},{ct}_{k},{ct}_{s},\hat{K})$
Game $G_{4}$**:**			
1.	${pk}_{k}\leftarrow U({PK}_{k})$		
2.	${pk}_{s}\leftarrow U({PK}_{s})$		
3.	${ct}_{k}\leftarrow U(k)$		
4.	${ct}_{s}\leftarrow U(C_{s})$		
5.	$\hat{K}\leftarrow U({\left\{0,\;1\right\}}^{\lambda })$		
6.	b←U({0, 1})		
7.	if b=0: $\;\;\;return\;({pk}_{k},{pk}_{s},{ct}_{k},{ct}_{s},K_{final})$ else: $\;\;\;return\;({pk}_{k},{pk}_{s},{ct}_{k},{ct}_{s},K_{final})$		

**Table 2:** Comparison of Security Features for Key Exchange Protocols in V2X Networks.

Security Objective	Kyber + Saber	RSA	ECDH
Post-Quantum Resistance	Strong	Weak	Weak
Man-in-the-Middle Resistance	Strong	Weak	Weak
Replay Attack Resistance	Strong	Weak	Weak
Forward Secrecy	Strong	Weak	Weak
Key Uniqueness and Entropy Quality	Strong	Moderate	Weak

**Table 3:** Virtual machine hardware and system configuration parameters.

Configuration Item	Parameter
Host Processo	Intel Core i5-14600KF
Virtual Machine Resources	2-core CPU, 4 GB RAM
Operating System	Debian 11
Simulation Framework and Toolchain	OMNeT++ 5.1, SUMO 1.8.0

**Table 4:** Key exchange protocol implementations and configurations.

Protocol	Algorithm and Parameters	Implementation Library and Version
RSA	RSA-2048	OpenSSL 3.0.7
ECDH	X25519	Libsodium 1.0.18
Kyber	Kyber512	PQClean optimized
Saber	LightSaber	PQClean optimized

**Table 5:** Evaluation metrics and measurement methodology.

Metric	Symbol	Unit	Definition/Measurement Scope
Handshake latency	$T_{HS}$	ms	Total elapsed time from OBU initiation to OBU verification, including KeyGen, Enc/Dec, KDF/HMAC, and network round-trip
Key generation time	$T_{kg}$	ms	Average duration of Kyber + Saber KeyGen phase
Encapsulation/Decapsulation time	$T_{enc}/T_{dec}$	ms	Combined time of Encaps + Decaps operations
KDF/HMAC time	$T_{kdf+hmac}$	ms	Time for HKDF key derivation + HMAC authentication computation
Message authentication delay	MAD	ms	HMAC generation/verification delay for a single vehicular message
CPU utilization	CPU%	%	Steady-state average/95th percentile
Memory usage	Mem	MB	Average/peak process RSS or working-set size
Total handshake traffic	$B_{HS}$	bytes	Total bytes transmitted + received in one handshake
Handshake success rate	HSR	%	Successful handshakes/total attempts

**Table 6:** Communication and Computational Complexity Analysis.

Field	Bytes
${ct}_{k}$	768
${ct}_{s}$	736
${ID}_{OBU}/{ID}_{RSU}$	4/4
${Nonce}_{OBU}/{Nonce}_{RSU}$	12/12
$Timestamp\;T_{1}/T_{2}$	4/4
${pk}_{id}$	16
HMAC	16

**Table 7:** Comparison of handshake latency and performance metrics among protocols.

Protocol	$T_{HS}\;(ms)$	$T_{kg}\;(ms)$	$T_{enc/dec}\;(ms)$	$T_{kdf+hmac}$	MAD (ms)	HSR (%)
Kyber + Saber	41.2 ± 2.8	12.6 ± 1.3	23.7 ± 1.9	4.9 ± 0.5	1.6 ± 0.3	98.2
ECDH	41.6 ± 3.2	22.5 ± 1.7	15.8 ± 1.4	3.3 ± 0.4	2.1 ± 0.4	96.7
RSA-2048	98.9 ± 4.7	80.6 ± 3.5	14.2 ± 1.6	4.1 ± 0.5	2.8 ± 0.6	92.3

**Table 8:** Comparison of resource utilization and communication overhead.

Protocol	CPU % (avg)	*Mem* (MB)	*Rounds*	$B_{HS}\;(bytes)$
Kyber + Saber	45.3	34.7	1.5	5200
ECDH	56.8	39.1	2	1000
RSA-2048	72.5	68.1	2	2400

**Table 9:** Overview of Core Security and Performance Features of the Kyber + Saber Protocol.

Core Requirement	Kyber + Saber Performance
Post-Quantum Resistance	Strong (Lattice-based cryptography)
Handshake Latency	Low (Single-round design, suitable for V2X)
Algorithm Adaptability	Lightweight, compatible with embedded systems
Authentication Mechanism	HMAC + Timestamp (Anti-replay, integrity verified)
Security Redundancy	Dual-path KEM encapsulation (Kyber + Saber)

## Data Availability

The data presented in this study are available on request from the corresponding author.
